# Toward Human-Centered Pedagogy in the Age of Generative AI Among Higher Education Educators: Qualitative Phenomenological Study

**DOI:** 10.2196/105172

**Published:** 2026-11-06

**Authors:** Zuheir Khlaif, Bilal Hamamra

**Affiliations:** 1 Learning and Education Leadership, United Arab Emirates University, Al Ain, Abu Dhabi, PS358, United Arab Emirates, 971 541466122

**Keywords:** generative AI, higher education, relational pedagogy, qualitative research, phenomenology

## Abstract

**Background:**

Generative AI (GenAI) expands possibilities for content generation, feedback, personalization, and instructional support in higher education. Its growing use also challenges established assumptions about teaching, assessment, professional expertise, academic integrity, and educational responsibility. Although research has examined technology adoption and AI competencies, less is known about how educators experience and interpret the pedagogical transformation associated with GenAI in everyday teaching.

**Objective:**

This study explored how higher education educators experience GenAI-mediated pedagogical transformation, focusing on pedagogical practices, professional roles and expertise, educator agency, human-AI relationships, and associated opportunities and tensions.

**Methods:**

A qualitative phenomenological inquiry involved 23 educators from 3 Palestinian universities. Purposive maximum-variation sampling sought variation in academic rank, discipline, teaching experience, and experience with AI tools. Individual semistructured, in-depth interviews were conducted from March to April 2026, audio-recorded, transcribed verbatim, and returned to participants for review. Data were analyzed using reflexive thematic analysis informed by phenomenology. Initial coding was inductive and grounded in participants’ accounts; human-centered AI and sociomateriality subsequently informed interpretation as sensitizing concepts. Themes were developed through iterative comparison of transcripts, analytic memos, and alternative interpretations. Informational sufficiency was observed after approximately 20 interviews, with 3 additional interviews used to examine the developing thematic structure.

**Results:**

The analysis generated 8 interconnected themes. Educators described a shift from content transmission toward mediation, inquiry, reflective learning, creative exploration, and process-oriented assessment. Professional expertise increasingly centered on facilitation, pedagogical design, contextual interpretation, ethical guidance, and evaluation of AI-generated content. Human-AI collaboration involved bounded and integrated configurations, with humans retaining responsibility for judgment and educational consequences. Educator agency appeared through decisions about AI participation, independent student reasoning, and the boundaries of human expertise. Credibility, bias, authorship, authenticity, and academic integrity became embedded as pedagogical concerns. Participants valued opportunities for creativity, personalization, flexibility, and instructional support while expressing concerns about dependence, reduced intellectual effort, and weakened human connection.

**Conclusions:**

GenAI-mediated pedagogical transformation depends on educators’ alignment of AI use with learning purposes, the allocation of responsibilities, and the preservation of ethical accountability and meaningful relationships. The resulting human-centered pedagogy framework positions pedagogical intentionality, contextual judgment, negotiated agency, and relational presence as central to responsible integration. Universities should complement technical training and institutional policies with support for pedagogical design, critical evaluation, process-oriented assessment, and ethical reasoning.

## Introduction

### Background

AI is rapidly transforming higher education, challenging traditional pedagogical paradigms and prompting educators to reconsider how teaching and learning are designed and enacted [1-3]. Recent advances in generative AI (GenAI), adaptive learning technologies, and intelligent tutoring systems have created new opportunities to personalize instruction, enhance feedback, and support data-informed educational decision-making [4,5]. However, integrating these technologies into educational practice also presents pedagogical, ethical, and professional challenges, requiring educators to continuously adapt their teaching approaches and decision-making processes [6,7].

GenAI now participates in knowledge creation, feedback, assessment, and curriculum work, placing educators at the point where technical capability becomes pedagogical practice [8,9]. Their decisions determine how AI-generated content is evaluated, contextualized, and integrated into student learning [6,10]. Although research has documented AI adoption, effectiveness, academic integrity, and implementation, less attention has been given to how educators interpret changing roles, authority, and responsibility in everyday teaching [4,11-13].

### AI in Higher Education: From Technological Innovation to Pedagogical Change

AI scholarship in higher education has often emphasized efficiency, personalization, and assessment, framing technology primarily as an instrument for improving established educational processes. GenAI complicates this view because it participates directly in explanation, drafting, feedback, and academic discourse, intervening in how knowledge is produced and authorized in settings defined by inquiry, authorship, and disciplinary judgment [5,11]. Unlike earlier digital tools that mainly organized or delivered learning, generative systems produce content during the pedagogical encounter. Their significance therefore arises from how generated material changes academic engagement, evaluation, and the distribution of intellectual work [7,14].

These capabilities create opportunities for adaptation and personalized support while simultaneously intensifying concerns about academic integrity, overreliance, mediation, and responsibility. Educators must, therefore, determine which activities may benefit from AI, which forms of judgment should remain human, and how educational value can be sustained when AI-generated content becomes part of knowledge production [8,15]. GenAI thus represents not merely another technological resource but a development that may require educators to reconsider the purposes, processes, and relationships underlying teaching and learning.

### Emerging Pedagogy in the AI Era

Emerging pedagogy concerns how teaching, knowledge mediation, and learning relationships are organized under conditions of AI participation. The central question is therefore not simply whether educators use AI, but how educational purposes and practices change when AI-generated content enters instruction, assessment, and feedback. The findings in the study [16] and [17] conceptualize this transformation through teacher positionality, rationality, and functionality, shifting attention from technical proficiency toward the roles and interactions through which authority, creativity, feedback, and meaning-making are organized.

The increasing availability of explanations, examples, and AI-generated content weakens information possession as the primary basis for teacher authority. Educators increasingly evaluate, contextualize, and direct AI outputs, placing discernment, relational guidance, and ethical interpretation at the center of pedagogical work [15]. Emerging AI-mediated pedagogy nevertheless remains contested. Creativity and flexibility coexist with concerns about authenticity, agency, judgment, and dependence, requiring educators to decide when to trust, challenge, limit, or redirect AI [9,11,18]. Consequently, pedagogical transformation in the AI era involves not only adopting new tools but also renegotiating how educational purposes, responsibilities, and relationships are enacted.

### Educators’ Lived Experiences in the AI Era

Competency models specify what educators should know and be able to do with AI, but they do not fully explain how teaching with AI is experienced within institutional expectations, disciplinary values, and changing professional identities. A phenomenological perspective therefore provides an important lens for examining uncertainty, adaptation, and the meanings educators construct through human-machine relations [3,17]. This focus is particularly important in higher education, where authorship, critique, originality, disciplinary judgment, and professional responsibility shape academic practice.

Examining educators’ lived experiences can reveal how they interpret and negotiate tensions involving authority, control, agency, and ethics as AI becomes embedded in routine teaching [13,16]. Such an approach moves beyond the questions of whether educators adopt AI or possess sufficient technical competencies and instead examines how AI-mediated practices are understood, experienced, and incorporated into educators’ professional identities and pedagogical decision-making.

### Theoretical Orientation: Human-Centered AI and Sociomateriality

Two complementary theoretical perspectives provide the conceptual orientation for interpreting educators’ experiences. First, human-centered AI emphasizes high levels of human control alongside capable automation and foregrounds self-efficacy, responsibility, safety, and trustworthiness [19]. Applied to higher education, this perspective focuses on educators’ judgment about when AI can support learning, where boundaries are needed, and how accountability remains human.

Second, sociomateriality treats practice as constituted through relations among people, technologies, institutional norms, and local contexts [20]. This perspective supports interpreting AI-mediated pedagogy as enacted through interconnected educator-student-AI relations rather than as an outcome of technology alone. Together, these perspectives provide complementary ways of understanding pedagogical transformation: human-centered AI foregrounds human judgment, responsibility, and control, whereas sociomateriality highlights the relational and contextual conditions through which AI-mediated practices are enacted. In the present study, both perspectives operated as sensitizing concepts during later interpretation rather than as a fixed deductive codebook; first-cycle coding remained grounded in participants’ accounts.

### Research Gap

Despite the growing body of scholarship on AI in higher education, an important gap remains concerning how educators experience and interpret pedagogical change after GenAI becomes part of routine teaching practice. Existing conceptual scholarship identifies changes in teacher positionality, classroom relations, authorship, agency, and ethical judgment [14-16,18], while empirical research has examined adoption, competencies, effectiveness, and implementation. However, qualitative evidence on how educators themselves make sense of these changes and enact them in everyday pedagogical practice remains limited.

In particular, insufficient attention has been given to how educators negotiate the relationship between AI affordances and human pedagogical judgment, how their professional roles and agency are reconstructed, and how they manage tensions between efficiency and educational purpose, technological assistance and human responsibility, and AI-supported activity and authentic learning. Addressing this gap requires attention to educators’ lived experiences and meaning-making rather than focusing exclusively on technological adoption or competency. Accordingly, this study adopts a phenomenologically informed approach to examine how higher education educators experience and interpret emerging pedagogy in the AI era.

### Study Purpose and Contribution

This study aimed to investigate higher education educators’ experiences of emerging pedagogy in the context of GenAI, with particular attention to changes in professional roles and agency, the ways educators exercise pedagogical judgment, and the opportunities and tensions associated with AI-mediated teaching. The inquiry therefore focused on relationships, judgment, and educational purpose, as well as technological tools and competencies [7,10]. An educator-centered interpretation of pedagogical change can clarify support needs relevant to faculty development, curriculum design, and institutional policy [21,22]. Understanding how educators negotiate AI-mediated teaching can also inform professional development that connects technical capability with critical evaluation, ethical judgment, and context-sensitive pedagogical design [17,23,24]. By focusing on educators’ lived experiences, this study seeks to contribute an empirically grounded understanding of how pedagogical purposes, professional roles, human-AI relationships, and educational responsibilities are being reconstructed as GenAI becomes increasingly embedded in higher education.

### Research Questions

The study addressed the following research questions:

1. How do higher education educators experience emerging pedagogy in the AI era?
2. How do higher education educators make sense of the influence of AI on their pedagogical roles, judgment, and agency?
3. How do educators manage the pedagogical opportunities and tensions that arise when teaching in AI-mediated higher education environments?

Accordingly, this study aimed to explore how higher education educators experience pedagogical transformation in the context of GenAI, with particular attention to changes in pedagogical roles, professional judgment, agency, human-AI relationships, and the opportunities and tensions associated with AI-mediated teaching.

## Methods

### Research Design

We used a qualitative phenomenological design to explore how higher education educators experienced and interpreted pedagogical transformation in the age of GenAI. Phenomenology guided the inquiry toward the meanings attributed to experience, while reflexive thematic analysis supported the interpretation of patterned meanings across participants’ accounts. This methodological combination enabled attention to individual meaning-making and shared experiential patterns without treating themes as objective entities discovered independently of the researchers’ interpretive work [25-27].

### Research Context and Participants

The study was conducted across 3 large comprehensive universities in Palestine that offer undergraduate and postgraduate programs in medicine and health sciences, engineering, education, business, the humanities, and other fields. Each institution had begun encouraging GenAI use through professional development or teaching initiatives, creating varied settings for examining educators’ experiences.

The study included 23 higher education educators with direct experience using GenAI in assessment design, feedback, course preparation, instructional support, or learning activities. Participants were recruited through purposive maximum-variation sampling [28,29]. Institutional contacts and professional academic networks circulated invitations to educators who met 2 inclusion criteria: employment in higher education and practical experience integrating GenAI into teaching. No invited participants declined participation or withdrew from the study. A demographic questionnaire asked participants to classify their experience as beginner, intermediate, or advanced according to the duration, frequency, and breadth of teaching-related AI use. These self-reported categories supported variation in the sample and did not represent a formal competency assessment.

Maximum-variation sampling captured shared and context-specific experiences across disciplines, ranks, teaching tenure, and self-reported AI experience. Table 1 summarizes the sample, and Appendix D in Multimedia Appendix 1 links P1 to P23 with disciplinary background, rank, teaching experience, and AI experience.

**Table 1.** Demographic characteristics of participants (N=23).

Variable and category	Frequency, n (%)
Gender	
Man	12 (52.2)
Woman	11 (47.8)
Academic rank	
Lecturer	6 (26.1)
Assistant professor	9 (39.1)
Associate professor	5 (21.7)
Professor	3 (13.0)
Years of teaching experience	
1‐5 years	5 (21.7)
6‐10 years	7 (30.4)
11‐15 years	6 (26.1)
More than 15 years	5 (21.7)
Discipline	
Humanities and social sciences	3 (13.0)
Education	5 (21.7)
Engineering and technology	4 (17.4)
Business and economics	4 (17.4)
Medical and health sciences	7 (30.4)
Experience with AI tools	
Beginner	5 (21.7)
Intermediate	11 (47.8)
Advanced	7 (30.4)

### Data Collection

Data collection was conducted from March to April 2026 through a single individual semistructured, in-depth interview with each participant. Both researchers were men faculty members with PhD degrees and had prior experience conducting qualitative research and teaching qualitative research courses in higher education. Both researchers conducted the interviews. Before the interviews, participants were informed about the researchers’ academic and research backgrounds, as well as the purpose of the study.

The interview guide used broad questions and flexible probes to elicit experiences, interpretations, and reflections. Literature on teacher positionality, pedagogical transformation, human-AI collaboration, professional agency, ethics, and human-centered AI informed the guide’s domains, while the wording remained open enough for participants to introduce unanticipated meanings. Before data collection, the interview guide was pilot-tested through a cognitive interview to assess the clarity, relevance, and comprehensibility of the questions. Appendix C in Multimedia Appendix 1 provides the interview protocol and representative questions.

Each interview lasted approximately 45 to 60 minutes. Face-to-face interviews were conducted at locations within the participants’ universities chosen by the participants, whereas a small number of participants preferred online interviews conducted in the evening. No individuals other than the participant and the researcher were present during the interviews. The study included no classroom observations and therefore used no observation protocol.

Before each interview, participants received information about the purpose of the study, voluntary participation, confidentiality, withdrawal rights, and audio recording, after which written consent was obtained. Interviews were audio-recorded with permission and transcribed verbatim. Field notes documented interview context and researchers’ preliminary reflections; they were not treated as observational data. Interview transcripts were returned to participants for review and to allow them to correct, add, or clarify their responses. No participants requested changes to their transcripts.

### Data Analysis

Interview data were analyzed using reflexive thematic analysis informed by the phenomenological orientation [25,26,30]. Repeated transcript reading supported familiarization, followed by first-cycle inductive coding of meaningful segments close to participants’ accounts. Both researchers independently coded the interview transcripts before discussing and refining the developing coding framework. Differences in coding and interpretation were addressed through reflexive discussion. The researchers did not use voting, calculate intercoder agreement, or designate one researcher as the final decision maker. When coding differences occurred, they returned to the relevant transcript and discussed the assumptions underlying each interpretation. They also considered other participants’ accounts, reflexive memos, and the study aim. This process led to clearer code definitions and the combination of overlapping codes. They retained distinct codes when they represented meaningfully different experiences. The researchers also revised the boundaries and descriptions of categories and themes when necessary. Differences in theme generation were handled through the same process. Each proposed theme was examined for internal coherence, distinctiveness, and support across the dataset. Decisions were documented in the audit trail. Competing interpretations were examined further until the researchers developed a shared interpretive account consistent with reflexive thematic analysis. Human-centered AI and sociomateriality supplied sensitizing concepts during later interpretation, but they did not determine the initial code set or impose themes in advance. Codes were iteratively compared and grouped into conceptual categories, which were refined into subthemes and overarching themes through recursive movement among transcripts, codes, memos, and developing interpretations. The team examined how participants connected pedagogical purpose, professional role, human-AI practice, agency, and ethics. Reflexive memos and an audit trail documented coding choices, alternative interpretations, category development, and theme revision. NVivo 15 (Lumivero, LLC) supported data organization, retrieval, and comparison. To enhance methodological transparency, Appendix A in Multimedia Appendix 1 illustrates the progression from interview excerpts to codes, categories, subthemes, and themes. Appendix B (Multimedia Appendix 1) presents the complete coding framework, and Appendix C (Multimedia Appendix 1) provides the semistructured interview protocol.

### Data Saturation

Data collection and analysis proceeded concurrently, allowing the team to assess informational sufficiency as the interpretation developed. After approximately 20 interviews, additional accounts no longer introduced substantively new meanings or conceptual relations and instead elaborated or qualified the existing analysis. This pattern approximated meaning saturation, as distinguished from early code saturation [31]. Sample adequacy was also considered through information power, including the focused aim, participants’ relevant experience, and the richness of the interviews [30,31]. Three additional interviews tested the stability and completeness of the developing thematic structure.

### Trustworthiness

We addressed credibility, transferability, dependability, and confirmability [32]. Credibility was supported through in-depth interviews, repeated transcript engagement, verbatim quotations linked to interpretive claims, and participant feedback. Preliminary themes were shared with participants who agreed to provide feedback, allowing them to comment on whether the interpretations reflected their experiences. An external qualitative researcher also independently coded approximately 10% of the transcripts using the developing framework. Differences in interpretation prompted analytic dialogue, clarification of code boundaries, and revision of the framework. This external review supported critical dialogue and was not used for intercoder reliability testing, consistent with the reflexive character of the analysis.

Transferability was supported through a detailed description of Palestine, the institutional setting, recruitment, inclusion criteria, participant characteristics, and maximum variation, allowing readers to assess relevance to other contexts. Dependability rested on an audit trail documenting recruitment, data collection, coding choices, category development, alternative interpretations, theme revision, and methodological decisions [28]. Appendix A in Multimedia Appendix 1 illustrates analytical progression, and Appendix B in Multimedia Appendix 1 presents the complete coding framework. Confirmability was supported through analytic memos, reflexive journals, transcript checks, and records of alternative interpretations. These materials connected the final account to participants’ words while acknowledging the researchers’ interpretive role.

### Researcher Reflexivity

Given the phenomenological orientation, the researchers maintained reflexivity throughout data collection, analysis, and interpretation [27,33]. Both researchers were men, faculty members with PhD degrees, and had prior experience conducting qualitative research and teaching qualitative research courses in higher education. Both researchers conducted the interviews. Before the interviews, participants were informed about the researchers’ academic and research backgrounds and the study’s purpose. The team comprised scholars with experience in educational technology, qualitative research, and GenAI in higher education. Collectively, they developed the protocol, conducted interviews, reviewed transcripts, coded data, developed themes, and examined the final interpretation. This background provided contextual familiarity and also created assumptions that required continuing scrutiny. Bracketing was practiced as an ongoing reflexive discipline, not as a claim that prior knowledge could be completely suspended. Interviewers recorded reflexive notes after data collection, and the team maintained journals, analytic memos, and an audit trail during coding and theme development. Regular discussions examined emerging interpretations against the transcripts and considered plausible alternatives. No prior supervisory, instructional, or evaluative relationships existed between researchers and participants, which reduced potential power asymmetries.

### Ethical Considerations

The study received ethical approval from the Research Department, Ministry of Higher Education (approval number March 2026/21). Before participating, all educators received information about the study’s purpose and procedures, the voluntary nature of participation, their right to decline to answer any question, and their right to withdraw from the study without penalty. We obtained written informed consent from all participants before conducting the interviews.

We protected participant privacy and confidentiality throughout the research process. Each participant was assigned a pseudonymous alphanumeric identifier (P1-P23), which was used in transcripts, data analysis, and reporting. Identifying information was removed from the research data and manuscript, and demographic and professional information was limited to details necessary for interpreting the findings to minimize the risk of deductive identification. Interview recordings and transcripts were stored securely and were accessible only to the research team. Participants received no financial or material compensation for their participation in the study.

### Reporting Guideline

This qualitative study was reported in accordance with the COREQ (Consolidated Criteria for Reporting Qualitative Research [34]), a 32-item checklist for qualitative studies involving interviews and focus groups. The completed COREQ checklist is provided as Checklist 1.

## Results

### Participant Characteristics

The study included 23 higher education educators from 3 Palestinian universities. The participants included 12 (52.2%) men and 11 (47.8%) women. Gender was self-reported by participants. No participants identified with a different gender category. Additional participant characteristics, including academic rank, teaching experience, discipline, and experience with AI tools, are presented in Table 1.

Following the presentation of participant characteristics, the analysis generated 8 interconnected themes describing educators' experiences of pedagogical transformation in the context of GenAI.

### Overview of Thematic Findings

Educators experienced GenAI as a reorganization in pedagogy, assessment, professional roles, and responsibilities. They identified possibilities for reflection, creativity, personalization, and innovation alongside uncertainty involving agency, authenticity, dependence, ethics, and human connection. The 8 themes represent interconnected dimensions of this experience.

#### Theme 1: Pedagogical Transformation

Participants located pedagogical change in knowledge mediation, assessment, and learning design. AI prompted the reconsideration of classroom interaction and educational purpose, not the routine addition of another tool.

##### From Transmission to Mediation

Educators reported moving away from transmission-based teaching toward a mediating role focused on helping students critically interpret and evaluate AI-generated material. As P1 explained:

*I am no longer just explaining content. I spend much more time helping students evaluate and use what AI gives them. Instead of providing immediate answers, I encourage them to question the AI’s responses, compare different sources, and think about why one answer may be more appropriate than another. I feel that my role has shifted from being the main source of knowledge to becoming someone who guides students in making informed judgments about the information they receive.*

[P1]

P1’s account locates pedagogical authority in guided evaluation. Asking students to question outputs, compare sources, and justify the suitability of an answer makes the educator responsible for cultivating judgment while students assume greater responsibility for evaluating information. Authority therefore shifts from possessing answers to orchestrating reasoned inquiry.

##### Process-Oriented Pedagogy

P4 described a growing emphasis on learning processes alongside final products:

*Now I care much more about how students used AI in developing their work than simply looking at the final answer. I want to understand the prompts they created, how they refined the responses, and why they accepted or rejected particular suggestions. Watching this process tells me much more about their learning than reading the finished assignment alone.*

[P4]

P4’s account recasts assessment as evidence of decision-making. Prompts, revisions, acceptances, rejections, and explanations made students’ reasoning visible and allowed the educator to evaluate how learning developed through interaction with AI.

##### Metacognitive and Reflective Pedagogy

P5 incorporated AI into activities that promoted students’ awareness of their own thinking:

*Whenever students submit AI-assisted work, I ask them to explain how they used the tool and what they decided to change. I want them to reflect on which ideas came from AI, which ideas were their own, and why they made certain decisions. These conversations help students realize that learning is not about accepting AI’s answers but about understanding their own thinking and improving it.*

[P5]

P5’s practice required students to distinguish generated suggestions from their own decisions and explain why they revised particular ideas. The AI-supported activity, therefore, became a prompt for metacognition when reflection and accountability were built into the task.

##### Fluid and Evolving Pedagogy

Participants also emphasized that pedagogy in the AI era remains dynamic and continually evolving. As P3 stated:

*This is not a fixed teaching model yet. Every semester I find myself changing my approach because both the technology and my students are changing. What worked a few months ago may no longer be effective, so I keep experimenting, reflecting, and adjusting my teaching. I think we are all still trying to understand what good teaching with AI really looks like.*

[P3]

P3’s account defines “good teaching with AI” as context-sensitive experimentation, reflection, and revision. The uncertainty does not indicate an absence of pedagogical purpose; it required continuing professional learning as technological capabilities and student practices change.

### Theme 2: Creative and Inquiry-Based Pedagogy

The second theme concerns inquiry, creativity, and authentic learning. Educators described AI as useful when it stimulated curiosity, supported exploration, and drew students into sustained engagement with complex questions.

#### Pedagogy of Wonder

Participants emphasized that the educational value of AI lay not in accelerating answers but in encouraging students to ask richer and more meaningful questions. As P2 explained:

*The value is not getting an answer faster, but using AI to push students toward better questions. I encourage my students to keep asking the AI different questions, challenge the responses, and explore alternative explanations instead of accepting the first answer they receive. When students become curious enough to continue questioning, I know the learning process has become much deeper than simply searching for information.*

[P2]

P2 evaluated AI through the quality of questions it enabled students to formulate. Educational value emerged when an initial response generated challenge, comparison, and further inquiry, so curiosity and critical exploration displaced answer speed as the measure of learning.

#### Iterative Inquiry

Participants also described AI-supported learning as an iterative process in which students continuously refined their understanding through repeated interaction with the technology. As P6 reflected:

*The process became more iterative. Students ask, revise, question, and go back again with AI. They rarely stop after the first response because every interaction leads to another question or another revision. I noticed that students became much more comfortable experimenting with different ideas and improving their work through repeated dialogue with the AI.*

[P6]

P6’s account presents learning as recursive: each response invites questioning, revision, and another attempt. Understanding develops through repeated evaluation and refinement instead of a fixed sequence from instruction to assessment.

#### AI-Enhanced Creativity

Educators treated generated suggestions as starting points whose value depended on student interpretation and transformation. As P11 explained:

*AI helps generate possibilities, but the real creativity comes from how students transform those ideas. I tell my students that AI can give them many starting points, but it cannot decide which ideas are meaningful or how they should be combined. Creativity happens when students evaluate, modify, and build on those suggestions to create something that reflects their own thinking.*

[P11]

Creativity, therefore, resided in selecting, combining, and reshaping possibilities into work that reflected the student’s judgment; generation alone did not constitute creative authorship.

#### Challenge- or Project-Based Pedagogy

Educators also designed authentic tasks that required students to apply AI within real-world problems. As P8 explained:

*I started designing tasks where students use AI to solve real problems instead of simply completing routine assignments. Rather than asking them to reproduce information, I ask them to investigate authentic situations, evaluate different solutions, and justify the decisions they make. AI becomes one resource within a much larger process of analysing and solving complex problems.*

[P8]

Project- and challenge-based tasks placed AI within analysis, justification, and problem solving. Technology supported inquiry while students retained ownership of decisions and outcomes.

### Theme 3: Repositioning the Educator’s Role

#### Overview

GenAI also altered how educators understood professional value. Facilitation, curation, ethical mentorship, design, and contextual judgment became central to their accounts of expertise.

Teacher positionality shift. Participants described moving away from seeing themselves as the primary source of knowledge toward becoming facilitators who help students assess increasingly complex information environments. As P7 explained:

*Students can get information anywhere now, so my role is more about guiding and framing their learning. Instead of spending most of my time delivering content, I focus on helping students decide which information is credible, how different ideas connect, and why certain sources are more trustworthy than others. I feel that my value as a teacher comes from helping students make sense of information rather than simply providing it.*

[P7]

P7 located authority in framing, connection, and credibility assessment. Expertise rested on helping students make sense of abundant information, not on controlling access to it.

#### Educator as a Curator

Participants also emphasized that AI-generated content required continuous human evaluation, adaptation, and contextualization before it could be integrated into teaching. As P10 reflected:

*AI can produce content quickly, but I still have to choose what is accurate and relevant for my course. Sometimes the information is incomplete, lacks the appropriate clinical context, or does not match my students' level of understanding. I spend a great deal of time reviewing, adapting, and reorganizing the material so that it supports my learning objectives rather than simply accepting what AI generates.*

[P10]

AI shifted professional effort toward selecting, refining, and contextualizing content. Curation, therefore, required disciplinary knowledge, awareness of student level, and alignment with learning objectives.

#### Educator as an Ethical Mediator

Beyond instructional responsibilities, participants described assuming a more explicit ethical role in helping students use AI responsibly. As P9 explained:

*I now spend more time discussing authorship, bias, and responsible AI use as part of teaching. Students often assume that if AI produces an answer, it must be correct, so we have conversations about transparency, academic integrity, and the importance of verifying information. These ethical discussions have become just as important as teaching the course content itself.*

[P9]

P9’s account places ethical guidance within routine higher education pedagogy. Transparency, verification, authorship, and bias become part of disciplinary learning, so technical competence cannot be separated from professional and societal responsibility.

#### Shifting Professional Identity

Participants further reflected on how AI had prompted them to reconsider their own professional identity and purpose as educators. As P15 stated:

*AI has forced me to rethink what makes me valuable as a teacher beyond delivering information. I realised that students need someone who can challenge their thinking, encourage reflection, and help them apply knowledge responsibly in real situations. The technology has changed the questions I ask myself about teaching, and in many ways it has strengthened my understanding of what my role should be.*

[P15]

P15 interpreted AI as a prompt to reconstruct their professional identity around challenge, reflection, responsible application, and meaningful learning relationships. The perceived source of value moved from content delivery to contextual and ethical guidance.

### Theme 4: Human-AI Relational Pedagogy

A fourth theme concerns the relational patterns through which educators organized AI-supported work. Participants used interpersonal metaphors to describe how AI influenced task development and instructional decision-making while retaining human accountability for interpretation and judgment.

#### Rationality With AI

P13 described AI as shaping interactions and task development: “It feels less like using software and more like working with a participant that keeps shaping the task.” This metaphor described the iterative influence of generated responses on pedagogical thinking; it did not attribute consciousness or moral agency to the system. Human educators remained responsible for evaluating and directing each contribution.

P16’s account locates educational value in the interpretive work through which educators and students evaluate, adapt, and refine generated material. Cocreation describes a process of joint activity without assigning moral agency to AI.

Bounded collaboration (“centaur” configuration). P14 described a divided arrangement in which AI-generated options were provided while humans retained final judgment: “I let AI generate options, but I keep the final judgment and evaluation for myself and the students.” The term identifies a workflow with explicit boundaries, where efficiency is accepted and interpretive responsibility remains human.

Integrated collaboration (“cyborg” configuration). P13 described continuous AI-assisted iteration across the teaching process: “Sometimes I work with AI back and forth through the whole teaching process, almost like an ongoing collaboration.” The term identifies an integrated workflow, not a fusion of human and machine agency. Continuous involvement increased the need for contextual evaluation and professional accountability.

### Theme 5: Negotiating Agency in AI-Mediated Teaching

Participants described agency as continual decisions about when, how, and to what extent AI should influence teaching and assessment. Technological assistance remained subordinate to educator-defined purposes and professional autonomy.

#### Retaining Pedagogical Control

P17 emphasized deliberate control over when and how AI entered teaching: “I use AI strategically, but I do not let it define the lesson or the assessment.” AI functioned as a resource for educator-defined purposes, preserving responsibility for learning objectives and assessment design.

P18’s account shows why contextual reading remained irreducibly human: classroom dynamics, student needs, and disciplinary expectations required situated interpretation. AI suggestions acquired pedagogical value only through that judgment.

P19 set boundaries according to the educational purpose of the task. Selective use of support protected independent reasoning and intellectual effort while allowing support where it strengthened learning.

#### Agency Under Pressure

P20 reported institutional and societal pressure to adopt AI because it was associated with innovation: “There is pressure to use AI because it is seen as progress, but I still need to decide what serves learning.” This account separates technological uptake from pedagogical improvement and locates responsible adoption in professional judgment.

### Theme 6: Critical and Ethical Pedagogy

Critical and ethical reasoning formed part of everyday AI-mediated pedagogy. Educators linked technical use to the capacity to assess reliability, bias, authorship, and consequence.

#### Bias and Accuracy Assessment

P21 treated critical evaluation as a learning outcome, requiring students to identify errors, biases, and contextual limits before using the generated material.

#### Algorithmic Literacy

P22 extended AI literacy beyond operation to include an understanding of how systems produce responses, where errors arise, and why evaluation remains necessary.

#### Academic Integrity and Authenticity

P4’s concern moved assessment toward evidence of reasoning, including prompts, revisions, and explanations, so authenticity could be evaluated through the process as well as the product.

#### Ethics as Pedagogy

P7’s account shows ethics entering classroom dialogue, assessment, and feedback as a guiding principle for responsible engagement with AI and its social consequences.

### Theme 7: Pedagogical Opportunities and Tensions

Educators evaluated AI through the interplay between pedagogical gains and educational costs. Efficiency, personalization, and access were weighed against dependence, reduced intellectual effort, and weakened human connection.

#### Efficiency in Planning and Feedback

P6 valued time savings when they released effort for discussion, individual support, and deeper teaching; efficiency mattered because of its pedagogical use.

#### Personalized and Differentiated Learning

P9 viewed AI as expanding the range of support available to students, while the educator remained responsible for adapting that support to individual needs.

#### Asynchronous and Ubiquitous Learning

P12 linked AI to learning beyond fixed classroom time and space: “Students can now continue engaging with course content and feedback outside the classroom in new ways.” This flexibility expands access to practice and feedback while increasing the need for clear expectations and educator guidance.

#### Overdependence on AI

P14’s concern identifies intellectual struggle as part of learning. Immediate generation could weaken independent thought when it displaces questioning and sustained engagement.

Loss of human connection. P18 treated dialogue, empathy, and trust as conditions of learning that technological support could not reproduce. Excessive reliance therefore risked distancing students from relational presence.

### Theme 8: Human-Centered Pedagogy

The final theme brings the preceding tensions into a human-centered orientation. Participants judged AI through empathy, responsibility, professional judgment, and the preservation of meaningful interaction.

#### Preserving Care and Presence

P2’s account defines care as responsiveness to students’ emotions, needs, and contexts. Technology could assist learning, but it could not assume this relational responsibility.

#### Meaningful Learning Beyond Efficiency

P11 distinguished productivity from educational quality. Speed mattered only when it supported reflection, critical thinking, and deep understanding.

#### Human Responsibility in Cocreated Learning

P16 retained human accountability for instructional design, ethical decisions, and the meaning of learning even when AI contributed to knowledge production.

### A Human-Centered Conceptual Framework for Emerging Pedagogy in the AI Era

We developed an empirically grounded interpretive framework comprising pedagogical transformation, educator repositioning, human-AI relational practice, negotiated agency, and critical-ethical pedagogy (Figure 1). Opportunities and tensions form the surrounding conditions, while human-centered pedagogy provides the organizing orientation. The synthesis arose from recurrent links across accounts and neither depends on code frequency nor claims causal validation.

**Figure 1.**
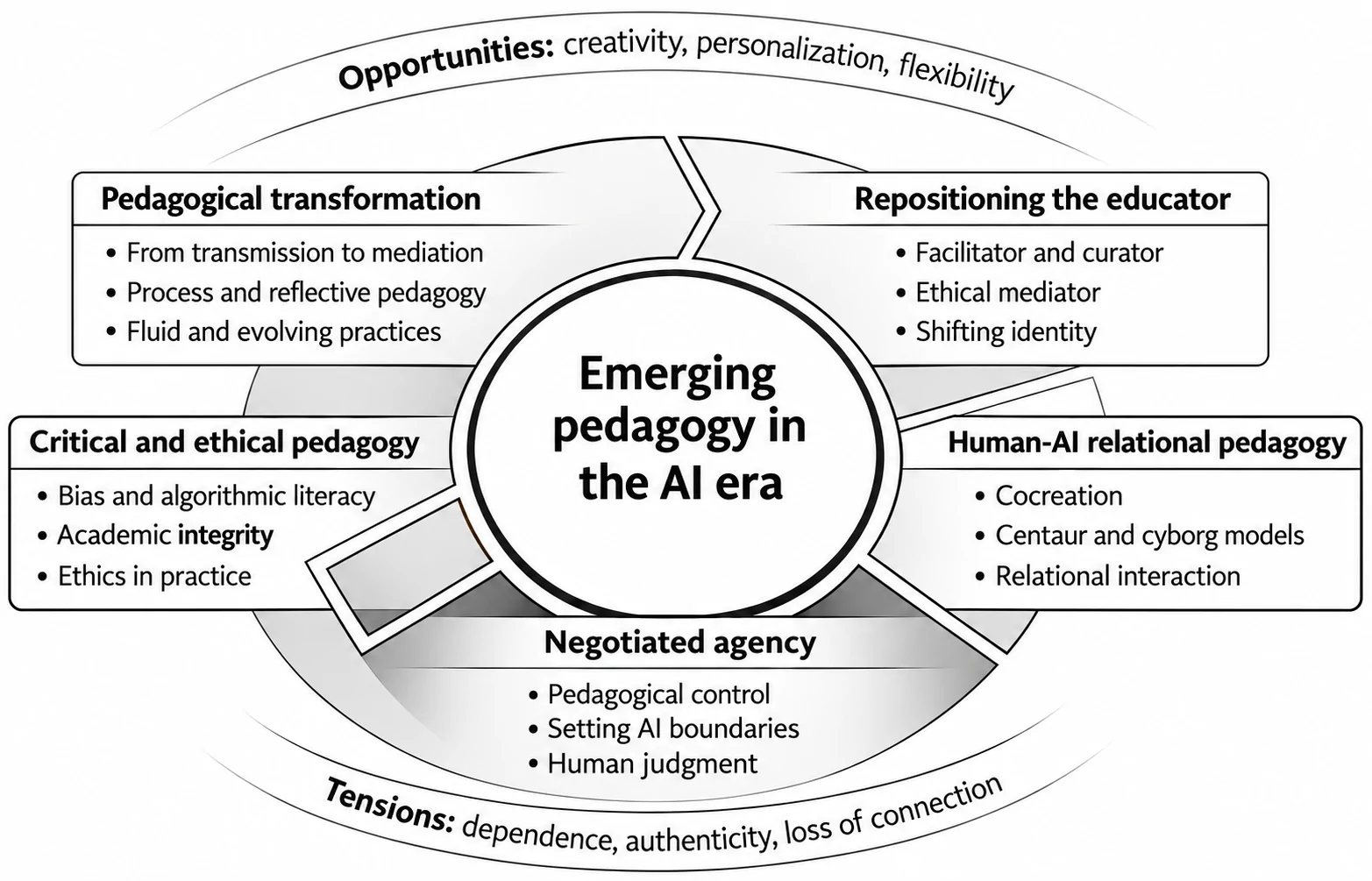
A human-centered conceptual framework for emerging pedagogy in the AI era.

The dimensions are recursive: shifts in educational purpose reshape professional roles; role reconstruction influences patterns of AI-supported work; these patterns prompt decisions about control and responsibility; and ethical judgment informs design, assessment, engagement, and boundaries.

Unlike adoption, literacy, digital competence, or instructional-function models, the framework interprets how pedagogy is reconstructed after AI enters everyday practice [3,15,25]. Human-centered AI clarifies control and accountability, while sociomateriality explains practice through relations among educators, students, systems, disciplines, and institutions [19,20].

## Discussion

### Principal Findings

This study explored how higher education educators experienced pedagogical transformation in the context of GenAI, focusing on changes in pedagogical practice, professional expertise, agency, human-AI relationships, and the opportunities and tensions associated with AI-mediated teaching. The findings show that educators experienced GenAI as more than an additional instructional technology. Its integration prompted them to reconsider how teaching, assessment, professional responsibility, and educational relationships should be organized. Participants described a movement from content transmission toward mediation, inquiry, reflection, creative exploration, and process-oriented assessment. They repositioned professional expertise around pedagogical design, contextual judgment, ethical guidance, and the allocation of responsibilities between humans and AI systems. Although they valued creativity, personalization, flexibility, and instructional support, they remained concerned about dependence, authenticity, academic integrity, weakened student effort, and reduced human interaction. Overall, the educational value of GenAI depended on how educators aligned its use with pedagogical purposes, ethical responsibilities, and the relational conditions of learning.

### Interpretation, Comparison With Previous Research, and Implications

The movement from content transmission toward inquiry, reflection, and evaluation indicates that pedagogical transformation cannot be understood solely as technology adoption. Previous research has highlighted the potential of GenAI to support personalization, feedback, creativity, and instructional efficiency [15,34]. The present findings qualify these claims by showing that technological affordances do not automatically produce meaningful learning. Participants designed activities that required students to interrogate AI-generated outputs, evaluate their credibility, revise them, and explain their decisions. They also made prompting, reasoning, and revision visible within the learning process. AI-generated content, therefore, became an object of critical examination rather than a finished academic product. Technological capability created possibilities for change, but educators’ intentional design and metacognitive guidance determined whether these possibilities contributed to learning [8,9,14].

This transformation also reconstructed professional expertise. As GenAI increasingly supported information retrieval, summarization, explanation, and content generation, participants located their professional value less in transmitting information and more in facilitation, curation, contextual interpretation, pedagogical design, and ethical mediation. This represents a more substantial change than adding technical competencies to an established teaching role [3]. AI-generated information still required interpretation within disciplinary, cultural, institutional, and classroom contexts. Educators’ authority therefore rested on recognizing students’ needs, guiding through uncertainty, evaluating AI-generated content, and connecting knowledge with disciplinary standards and educational purposes. Technical competence acquired educational value only when combined with pedagogical, relational, and interpretive expertise.

The findings further clarify the relational character of human-AI collaboration. Participants described bounded collaboration, in which AI generated ideas or alternatives while humans retained explicit evaluative authority, and integrated collaboration, in which AI-assisted iteration continued throughout a task. Both configurations could support questioning, idea development, and revision [7,14]. Neither configuration, however, was inherently pedagogically effective. Their value depended on how educators established boundaries, distributed responsibilities, interpreted outputs, and maintained accountability. From a sociomaterial perspective, learning emerged through relationships among educators, students, AI systems, disciplinary expectations, assessment practices, and institutional conditions rather than through the technology alone [14]. Consequently, the same AI function could support inquiry in one context but encourage dependence or superficial engagement in another.

Educator agency was similarly expressed through professional discretion rather than a binary choice between adoption and resistance. Participants decided when AI should contribute, which activities required independent student reasoning, how AI-generated outputs should be evaluated, and where human expertise needed to remain central. This interpretation extends beyond adoption models, which can explain why educators use technology but offer less insight into how they allocate cognitive, pedagogical, and ethical responsibilities after adoption. Consistent with human-centered AI, the findings position professional judgment as the mechanism through which technological capabilities are combined with meaningful human control and accountability [19].

Ethical reasoning was embedded within pedagogical practice rather than treated solely as an external regulatory requirement. Previous scholarship has commonly addressed AI ethics through governance, privacy, academic integrity, transparency, and technological risk [18]. Participants incorporated these concerns into teaching and assessment by asking students to examine credibility, identify bias, consider authorship, disclose AI use, and evaluate the consequences of relying on AI-generated content. AI literacy therefore extended beyond operational competence to include critical evaluation, intellectual responsibility, and informed judgment [35]. This suggests that responsible AI use cannot be developed through institutional rules alone. It must also be cultivated through instructional activities, assessment, dialogue, and feedback.

Participants’ simultaneous recognition of opportunities and concerns should not be interpreted as inconsistency. They valued AI’s capacity to support creativity, personalization, flexibility, and instructional assistance while expressing concern about dependence, weakened effort, reduced authenticity, and diminished human interaction [4,35]. These positions reflected evaluations of different pedagogical consequences. AI use was viewed positively when it supported inquiry, critical thinking, student agency, and meaningful engagement. It was viewed more cautiously when it replaced reasoning, obscured authorship, or weakened educator-student interaction. Educators’ attitudes were therefore conditional on the purposes, boundaries, and relationships associated with particular uses of AI.

The human-centered pedagogy framework integrates these findings by connecting pedagogical transformation, educator repositioning, human-AI relational practice, negotiated agency, and critical-ethical pedagogy. It extends models centered primarily on technology adoption, competence, or instructional function [18,34,36]. The framework identifies pedagogical intentionality as the connection between technological affordances and educational value, contextual judgment as a foundation of professional expertise, responsibility allocation as an expression of agency, and ethical reasoning as part of pedagogy. These dimensions are recursive: changes in assessment reshape educators’ roles, reconstructed roles influence how tasks are distributed between humans and AI, and these arrangements generate further decisions about transparency, accountability, and oversight.

The framework also demonstrates the complementary contributions of human-centered AI and sociomateriality. Human-centered AI explains the continuing importance of professional judgment, responsibility, oversight, and appropriate boundaries. Sociomateriality explains how these decisions are enacted through relationships among educators, students, AI systems, disciplinary practices, and institutional conditions [20,22]. Pedagogical agency is therefore neither located entirely within educators nor transferred to technology. It is exercised through how educators organize human and technological contributions while retaining responsibility for their educational consequences. Because the framework was developed interpretively from educators’ accounts, it remains open to refinement and empirical examination in other disciplines, institutions, and cultural settings.

These findings have practical implications for faculty development, curriculum, assessment, and institutional policy. Professional development should connect technical knowledge with learning design, process-oriented assessment, critical evaluation, ethical reasoning, and responsible human-AI collaboration. Educators need opportunities to design activities that make students’ prompting, reasoning, verification, revision, and justification visible. They also need support in determining when AI enhances learning and when it replaces the intellectual effort or interpersonal engagement an activity is intended to develop. Professional judgment should therefore be treated as a central capacity for AI integration rather than as an outcome that follows automatically from technical competence.

Universities should also translate general principles concerning bias, credibility, authorship, authenticity, transparency, and academic integrity into classroom and assessment practices. Ethical guidance becomes educationally meaningful when students apply it while evaluating AI-generated content and explaining their decisions. Institutional policies should establish shared expectations for responsibility and transparency while preserving educators’ capacity to make context-sensitive decisions. Uniformly restrictive approaches may prevent valuable experimentation, whereas overly permissive policies may weaken academic standards and leave educators without sufficient guidance. Finally, universities should ensure that efficiency and personalization do not displace interaction, mentorship, care, and educator presence. AI integration should be evaluated not only by what it automates but also by whether it strengthens pedagogical quality, student agency, ethical responsibility, and meaningful human relationships.

### Limitations and Future Research

This study should be interpreted in light of several limitations. The findings were derived from interviews with 23 educators across 3 Palestinian universities. The qualitative design supported an in-depth examination of lived experiences and professional judgment, but the findings are contextually situated and are not intended to be statistically generalized to all higher education institutions. Institutional policies, technological infrastructure, disciplinary traditions, and sociocultural expectations may shape AI-mediated pedagogy differently elsewhere. The contextual detail and variation within the sample may nevertheless help readers assess the transferability of the findings to comparable settings.

The study focused on educators’ accounts and did not include students, institutional leaders, or policymakers. It therefore explains how educators understood pedagogical transformation but cannot establish whether other stakeholders experienced or interpreted AI-supported practices similarly. Future research should incorporate multiple perspectives and examine where understandings of responsible AI use converge or differ. Research involving students could investigate metacognition, creativity, critical AI literacy, academic integrity, dependence, and perceptions of educator presence.

The findings were also based on reported experiences rather than direct observations of classroom practices or analyses of AI-supported learning activities. Participants’ accounts provided access to the interpretations and professional reasoning central to the study’s aim, but they may not fully represent how pedagogical decisions are enacted. Future studies could combine interviews with classroom observations, assessment tasks, student work, AI interaction records, or institutional documents to examine relationships between stated pedagogical intentions and enacted practices.

GenAI technologies, institutional policies, and educational practices are developing rapidly. The findings, therefore, represent a particular period within an ongoing transition. Longitudinal research is needed to examine how professional roles, pedagogical judgment, and the allocation of human-AI responsibilities evolve. Finally, the proposed framework is interpretive rather than predictive or causal. Comparative, mixed methods, and quantitative studies could refine and examine relationships among its dimensions across disciplines, institutions, and cultural contexts.

### Conclusions

The growing presence of GenAI in higher education requires more than the adoption of new tools or the development of technical competencies. It requires universities to reconsider how educational purpose, professional authority, student agency, and responsibility are defined when teaching and knowledge production increasingly involve AI systems. The central challenge is not simply whether GenAI should be used but how its use can be organized so that technological capabilities strengthen rather than displace critical thinking, meaningful assessment, ethical responsibility, and human relationships.

The human-centered pedagogy framework provides a foundation for addressing this challenge by placing pedagogical intentionality, contextual judgment, negotiated responsibility, and relational engagement at the center of AI-supported education. Responsible integration cannot be achieved through technical training, regulation, or access to technology alone. Universities must enable educators and students to question AI-generated outputs, justify decisions, establish appropriate boundaries, and remain accountable for the educational consequences of AI use. As GenAI continues to evolve, the quality of higher education will depend not only on what these systems can produce but also on whether institutions preserve the professional judgment, student agency, ethical reasoning, and human connection that give education its purpose.

## Supplementary material

10.2196/105172 Multimedia Appendix 1 Coding book for analyzing higher education educators’ lived experiences of emerging pedagogy in the AI era.

10.2196/105172 Checklist 1 COREQ checklist.

## Abbreviations

COREQ: Consolidated Criteria for Reporting Qualitative Research
GenAI: generative AI
