# Supplementary material for: Toward Human-Centered Pedagogy in the Age of Generative AI Among Higher Education Educators: Qualitative Phenomenological Study
Source: JMIR Med Educ. 2026 Nov 6;12:e105172. doi: 10.2196/105172 (PMC13742774; doi:10.2196/105172)
Supplement: Supplementary file 1 [file mededu-v12-e105172-s002.pdf]

COREQ (COnsolidated criteria for REporting Qualitative research) Checklist

Updated to reflect the revised manuscript and the authors' confirmed methodological details.

Topic	Item No.	Guide Questions/Description	Reported in Manuscript
**Domain 1: Research team and reflexivity**			
Interviewer/facilitator	1	Which author/s conducted the interview or focus group?	Researcher Reflexivity: Both researchers conducted the interviews.
Credentials	2	What were the researcher's credentials?	Researcher Reflexivity: Both researchers held PhD degrees.
Occupation	3	What was their occupation at the time of the study?	Researcher Reflexivity: Both researchers were faculty members.
Gender	4	Was the researcher male or female?	Researcher Reflexivity: Both researchers were male.
Experience and training	5	What experience or training did the researcher have?	Researcher Reflexivity: Both had prior qualitative research experience and had taught qualitative research courses in higher education.
Relationship established	6	Was a relationship established prior to study commencement?	Researcher Reflexivity: No prior supervisory, instructional, or evaluative relationships existed.
Participant knowledge of interviewer	7	What did participants know about the researcher?	Researcher Reflexivity: Participants were informed about the researchers' academic/research backgrounds and study purpose.
Interviewer characteristics	8	Bias, assumptions, reasons and interests in the research topic	Researcher Reflexivity: Assumptions, bracketing, reflexive notes, journals, memos, and audit trail reported.
**Domain 2: Study design**			
Methodological orientation and theory	9	What methodological orientation underpinned the study?	Research Design/Data Analysis: Qualitative phenomenological design with reflexive thematic analysis.
Sampling	10	How were participants selected?	Research Context and Participants: Purposive maximum variation sampling.
Method of approach	11	How were participants approached?	Research Context and Participants: Invitations circulated through institutional contacts and professional academic networks.
Sample size	12	How many participants were in the study?	Research Context and Participants/Results: N=23.
Non-participation	13	How many refused or dropped out? Reasons?	Research Context and Participants: No invited participants declined participation or withdrew.
Setting of data collection	14	Where was the data collected?	Data Collection: Face-to-face interviews at participant-selected locations within their universities; a small number preferred online evening interviews.
Presence of non-participants	15	Was anyone else present besides participants and researchers?	Data Collection: No individuals other than the participant and researcher were present.
Description of sample	16	Important characteristics of the sample	Results, Participant Characteristics, Table 1.
Interview guide	17	Were questions/prompts/guides provided? Was it pilot tested?	Data Collection and Appendix C: Semistructured guide; pilot-tested through a cognitive interview for clarity, relevance, and comprehensibility.
Repeat interviews	18	Were repeat interviews carried out?	Data Collection: One individual interview was conducted with each participant; no repeat interviews.
Audio/visual recording	19	Did the research use audio or visual recording?	Data Collection: Interviews were audio-recorded with permission.
Field notes	20	Were field notes made during/after interviews?	Data Collection: Field notes documented interview context and preliminary reflections.
Duration	21	Duration of interviews	Data Collection: Approximately 45-60 minutes.
Data saturation	22	Was data saturation discussed?	Data Saturation: Saturation was assessed concurrently; no substantively new themes after interview 20, followed by three confirmatory interviews.
Transcripts returned	23	Were transcripts returned to participants for comment/correction?	Data Collection: Transcripts were returned for review, correction, addition, or clarification; no participants requested changes.
**Domain 3: Analysis and findings**			
Number of data coders	24	How many data coders coded the data?	Data Analysis: Both researchers independently coded the transcripts before discussion and refinement.

Topic	Item No.	Guide Questions/Description	Reported in Manuscript
Description of coding tree	25	Did authors provide a description of the coding tree?	Data Analysis; Appendices A-B: Progression from excerpts to codes, categories, subthemes, and themes is provided.
Derivation of themes	26	Were themes identified in advance or derived from the data?	Data Analysis: First-cycle coding was inductive; theoretical lenses were later sensitizing concepts and did not impose themes.
Software	27	What software was used to manage the data?	Data Analysis: NVivo 15.
Participant checking	28	Did participants provide feedback on the findings?	Trustworthiness: Preliminary themes were shared with participants who agreed to provide feedback; an external qualitative expert also independently coded ~10% of the data.
Quotations presented	29	Were participant quotations presented and identified?	Results: Participant quotations are presented and identified using P1-P23.
Data and findings consistent	30	Was there consistency between data presented and findings?	Results: Interpretive claims are supported with participant quotations across themes.
Clarity of major themes	31	Were major themes clearly presented?	Results: Eight major themes are clearly presented.
Clarity of minor themes	32	Were diverse cases/minor themes described?	Results: Subthemes, tensions, and variation in participant experiences are described.

COREQ source: Tong A, Sainsbury P, Craig J. Consolidated criteria for reporting qualitative research (COREQ): a 32-item checklist for interviews and focus groups. *Int J Qual Health Care*. 2007;19(6):349-357.

Note: This checklist uses manuscript section locations rather than fixed page numbers because pagination may change during final JMIR formatting. Page numbers can be inserted after the final manuscript layout is fixed.
