# Supplementary material for: Toward Human-Centered Pedagogy in the Age of Generative AI Among Higher Education Educators: Qualitative Phenomenological Study
Source: JMIR Med Educ. 2026 Nov 6;12:e105172. doi: 10.2196/105172 (PMC13742774; doi:10.2196/105172)
Supplement: Supplementary file 2 [file mededu-v12-e105172-s001.docx]

**Appendixes**

**Appendix A**

### Appendix A. Example of the Coding Process

The following excerpt illustrates how a representative segment of interview data was progressively transformed into first-cycle codes, conceptual categories, subthemes, and final themes during the analytical process.

| **Representative Interview Excerpt** | **Initial Code (First-Cycle Coding)** | **Conceptual Category** | **Subtheme** | **Main Theme** |
| --- | --- | --- | --- | --- |
| "I am no longer just explaining content. I spend more time helping students evaluate and use what AI gives them." *(P1)* | Moving beyond explaining content; Helping students evaluate AI | Teaching as guidance rather than information delivery | **From transmission to mediation** | **Emerging Pedagogy as Pedagogical Transformation** |
| "Now I care more about how students used AI in developing the work, not just the final answer." *(P2)* | Focusing on how students learn with AI | Learning process over final product | **Process-oriented pedagogy** | **Emerging Pedagogy as Pedagogical Transformation** |
| "I ask students to explain how they used AI and what they changed, so they become aware of their own thinking." *(P3)* | Encouraging students to reflect on AI use | Reflection and self-monitoring | **Metacognitive and reflective pedagogy** | **Emerging Pedagogy as Pedagogical Transformation** |
| "Students can get information anywhere now, so my role is more about guiding and framing their learning." *(P9)* | My role is changing from teaching to guiding | Changing educator responsibilities | **Teacher positionality shift** | **Repositioning the Educator's Role** |
| "AI has forced me to rethink what makes me valuable as a teacher beyond delivering information." *(P12)* | Rethinking my value as a teacher | Professional identity reconstruction | **Shifting professional identity** | **Repositioning the Educator's Role** |
| "It feels less like using software and more like working with a participant that keeps shaping the task." *(P13)* | AI feels like a participant | AI as an active teaching partner | **Relationality with AI** | **Human–AI Relational Pedagogy** |
| "The work is no longer purely human or purely AI; it is something co-created in the process." *(P14)* | Shared creation with AI | Collaborative knowledge construction | **Human–AI co-creation** | **Human–AI Relational Pedagogy** |
| "I let AI generate options, but I keep the final judgment and evaluation for myself and the students." *(P15)* | Humans keep final judgment | Human oversight of AI | **Centaur model** | **Human–AI Relational Pedagogy** |
| "Teaching is still about human connection, and I worry that too much AI can distance students from that." *(P18)* | Teaching depends on human connection | Preserving human relationships | **Preserving care and presence** | **Human-Centered Pedagogy in the AI Era** |
| "My concern is not whether AI makes things faster, but whether it still supports meaningful learning." *(P11)* | Meaning matters more than efficiency | Prioritising meaningful learning | **Meaningful learning beyond efficiency** | **Human-Centered Pedagogy in the AI Era** |
| "AI can contribute to the process, but responsibility for the learning still remains human." *(P16)* | Humans remain responsible for learning | Human responsibility in AI-supported learning | **Human responsibility in co-created learning** | **Human-Centered Pedagogy in the AI Era** |

**Note.** During analysis, interview transcripts were first segmented into meaningful units and assigned descriptive first-cycle codes that remained close to participants' language. Similar codes were then grouped into conceptual categories through iterative comparison across the dataset. These categories were subsequently refined into subthemes and integrated into the overarching themes presented in the findings. The table provides an illustrative audit trail of this analytical progression rather than a complete coding record.

**Appendix B**

**Table 2**

Table 2. Coding Book for the Analysis of Higher Education Educators’ Lived Experiences of the Emerging Pedagogy of the AI Era

| **Main Theme** | **Subtheme** | **Definition of Subtheme** | **Example Quotations from Participants** | **Study Question** | **Coding Approach** |
| --- | --- | --- | --- | --- | --- |
| **Emerging Pedagogy as Pedagogical Transformation** | From transmission to mediation | Teaching shifts from delivering information to guiding, filtering, and contextualizing AI-supported knowledge. | “I am no longer just explaining content. I spend more time helping students evaluate and use what AI gives them” (P1). | RQ1 | Hybrid (Literature-informed & refined inductively) |
|  | Process-oriented pedagogy | Educators emphasize learning processes, including how students think, revise, and interact with AI, rather than only final products. | “Now I care more about how students used AI in developing the work, not just the final answer” (P2). | RQ1/RQ3 | Hybrid |
|  | Metacognitive and reflective pedagogy | Educators encourage students to reflect on their own thinking, monitor their use of AI, and evaluate AI-generated output critically. | “I ask students to explain how they used AI and what they changed, so they become aware of their own thinking” (P3). | RQ1/RQ3 | Deductive (Literature-informed) |
|  | Fluid and evolving pedagogy | Pedagogy is experienced as dynamic, unsettled, and continuously developing in response to AI. | “This is not a fixed teaching model yet. We are still redefining what good teaching means with AI” (P4). | RQ1 | Inductive (Data-driven) |
| **Creative and Inquiry-Based Pedagogy** | Pedagogy of wonder | AI is used to stimulate deeper questioning, curiosity, and exploration rather than only quick answers. | “The value is not getting an answer faster, but using AI to push students toward better questions” (P5). | RQ1/RQ3 | Inductive |
|  | Iterative inquiry | Students and educators use AI interactively through repeated prompting, refinement, and exploration of multiple perspectives. | “The process became more iterative. Students ask, revise, question, and go back again with AI” (P6). | RQ1/RQ3 | Hybrid |
|  | AI-enhanced creativity | AI supports brainstorming, ideation, prototyping, and innovation while human judgment refines the output. | “AI helps generate possibilities, but the real creativity comes from how students transform those ideas” (P7). | RQ3 | Hybrid |
|  | Challenge- or project-based pedagogy | AI is integrated into authentic tasks and real-world problem solving rather than isolated classroom exercises. | “I started designing tasks where students use AI to solve real problems, not just complete routine assignments” (P8). | RQ1/RQ3 | Deductive |
| **Repositioning the Educator's Role** | Teacher positionality shift | Educators move from authoritative knowledge-holder to facilitator, co-learner, mediator, or guide. | “Students can get information anywhere now, so my role is more about guiding and framing their learning” (P9). | RQ2 | Hybrid |
|  | Educator as curator | Educators select, verify, adapt, and refine AI-generated content for pedagogical use. | “AI can produce content quickly, but I still have to choose what is accurate and relevant for my course” (P10). | RQ2 | Inductive |
|  | Educator as ethical mediator | Educators guide students in responsible, critical, and ethical AI use. | “I now spend more time discussing authorship, bias, and responsible AI use as part of teaching” (P11). | RQ2/RQ3 | Deductive |
|  | Shifting professional identity | Educators reflect on how AI changes their self-understanding as teachers in higher education. | “AI has forced me to rethink what makes me valuable as a teacher beyond delivering information” (P12). | RQ2 | Inductive |
| **Human–AI Relational Pedagogy** | Relationality with AI | AI is experienced not merely as a tool, but as an active conversational presence in teaching and learning. | “It feels less like using software and more like working with a participant that keeps shaping the task” (P13). | RQ1/RQ2 | Inductive |
|  | Human–AI co-creation | Teaching and learning involve shared production between humans and AI. | “The work is no longer purely human or purely AI; it is something co-created in the process” (P14). | RQ1/RQ2 | Inductive |
|  | Centaur model | Humans retain judgment while AI supports idea generation and processing. | “I let AI generate options, but I keep the final judgment and evaluation for myself and the students” (P15). | RQ2/RQ3 | Hybrid |
|  | Cyborg model | Educators and AI work collaboratively through continuous interaction. | “Sometimes I work with AI back and forth through the whole teaching process, almost like an ongoing collaboration” (P16). | RQ2/RQ3 | Hybrid |
| **Negotiating Agency in AI-Mediated Teaching** | Retaining pedagogical control | Educators actively decide when and how AI should be used. | “I use AI strategically, but I do not let it define the lesson or the assessment” (P17). | RQ2 | Inductive |
|  | Human judgment over automation | Pedagogical decisions require human interpretation and contextual awareness. | “AI can suggest many things, but it cannot read the classroom context like I can” (P18). | RQ2 | Hybrid |
|  | Boundaries of AI use | Educators establish limits for AI use to preserve learning quality. | “There are tasks where I allow AI and others where I draw a clear line because students need to think independently” (P19). | RQ2/RQ3 | Inductive |
|  | Agency under pressure | Educators negotiate institutional expectations while maintaining pedagogical autonomy. | “There is pressure to use AI because it is seen as progress, but I still need to decide what serves learning” (P20). | RQ2/RQ3 | Inductive |
| **Critical and Ethical Pedagogy in the AI Era** | Bias and accuracy assessment | Students learn to critically evaluate AI-generated outputs. | “Students need to learn that AI outputs are not neutral and must be checked carefully” (P21). | RQ3 | Deductive |
|  | Algorithmic literacy | Understanding how AI systems function and their limitations. | “I want students not only to use AI, but to understand what kind of system they are dealing with” (P22). | RQ3 | Deductive |
|  | Academic integrity and authenticity | Concerns regarding originality and authentic student learning. | “The challenge is knowing whether the assignment reflects student thinking or just smart prompting” (P4). | RQ3 | Deductive |
|  | Ethics as pedagogy | Ethical reflection becomes embedded within everyday teaching practice. | “Ethics is no longer an extra topic. It has become part of the teaching process itself” (P7). | RQ2/RQ3 | Hybrid |
| **Pedagogical Opportunities and Tensions** | Efficiency in planning and feedback | AI supports planning and feedback processes. | “AI saves me time in preparing examples and feedback, which gives me more time for deeper teaching” (P6). | RQ3 | Deductive |
|  | Personalized and differentiated learning | AI facilitates individualized learning support. | “It helps me respond better to student differences because I can provide more varied support” (P9). | RQ3 | Deductive |
|  | Asynchronous and ubiquitous learning | Learning extends beyond classroom boundaries. | “Students can now continue engaging with course content and feedback outside the classroom in new ways” (P12). | RQ1/RQ3 | Deductive |
|  | Overdependence on AI | Concerns regarding excessive reliance on AI. | “Some students stop wrestling with ideas once they know AI can produce something instantly” (P14). | RQ3 | Deductive |
|  | Loss of human connection | Concerns about reduced interpersonal interaction. | “Teaching is still about human connection, and I worry that too much AI can distance students from that” (P18). | RQ3 | Inductive |
| **Human-Centered Pedagogy in the AI Era** | Preserving care and presence | Empathy and human responsiveness remain central to teaching. | “Students still need a teacher who understands them as people, not just a system that generates responses” (P2). | RQ2/RQ3 | Hybrid |
|  | Meaningful learning beyond efficiency | Learning quality is prioritized over technological efficiency. | “My concern is not whether AI makes things faster, but whether it still supports meaningful learning” (P11). | RQ1/RQ3 | Inductive |
|  | Human responsibility in co-created learning | Humans remain responsible for educational judgment and meaning-making. | “AI can contribute to the process, but responsibility for the learning still remains human” (P16). | RQ2/RQ3 | Hybrid |

### **Note.**

The analysis followed a **hybrid reflexive thematic analysis**. Deductive codes were informed by the literature on generative AI, pedagogy, educator roles, ethics, and human-centered learning, serving as **sensitizing concepts** rather than predetermined categories. Inductive codes emerged through iterative engagement with participants' narratives. Codes identified as **Hybrid** represent concepts that were initially informed by the literature but were substantially refined and reinterpreted through participants' lived experiences during the analytical process.

## Appendix C. Interview Protocol and Semi-Structured Interview Guide

**Dear Participant,**

Thank you for agreeing to participate in this study. The purpose of this research is to explore how higher education educators experience and interpret pedagogical change in the age of generative artificial intelligence (AI). We are interested in understanding your experiences, perspectives, and reflections on teaching and learning in this evolving educational context.

You have been invited to participate because you are a higher education educator with experience in integrating or engaging with generative AI in your teaching or related educational practices. Your experiences and insights are valuable for helping us better understand how educators are responding to the opportunities and challenges associated with generative AI in higher education.

The interview is expected to last approximately **45–60 minutes**. There is no financial compensation for participating in this study. Your participation is entirely voluntary, and you may choose to withdraw from the study at any time without providing a reason or facing any consequences. You are also free to decline to answer any question that you feel uncomfortable answering.

With your permission, the interview will be audio-recorded to ensure an accurate record of your responses. All recordings will be transcribed and anonymized before analysis. Your name and any identifying information will be removed from the transcripts, and no information that could identify you will appear in any publications or reports resulting from this study. All data collected will be treated confidentially, stored securely, and used solely for scientific research purposes by the research team.

There are no right or wrong answers. We are interested in your personal experiences and perspectives. Please feel free to speak openly and provide examples whenever you consider them relevant. If you have any questions before we begin, I will be happy to answer them.

**Do you have any questions before we start? May I begin the interview and start the audio recording?**

| **No.** | **Interview Question** | **Follow-up Probes** |
| --- | --- | --- |
| 1 | Can you describe your experiences of teaching since generative AI became widely available? | What stands out most? Can you recall a particular experience? |
| 2 | In what ways, if any, has generative AI influenced your teaching practices? | Can you provide an example? How did you respond? |
| 3 | How has the increasing availability of generative AI influenced your interactions with students? | Has anything changed in communication, feedback, or classroom engagement? |
| 4 | Can you describe how, if at all, your role as an educator has changed while teaching in the age of generative AI? | What aspects of your role feel different? What remains unchanged? |
| 5 | What opportunities have you experienced from using or teaching alongside generative AI? | Can you describe situations where AI supported teaching or learning? |
| 6 | What challenges or concerns, if any, have you encountered while teaching in an environment where students have access to generative AI? | How have you addressed these situations? |
| 7 | How do you approach assessment and student learning when generative AI tools are available? | Have your assessment or feedback practices changed? Can you give an example? |
| 8 | Looking back on your experiences, how would you describe the overall impact of generative AI on your teaching and professional practice? | What has been most significant for you? Why? |
| 9 | Is there anything about your experience of teaching in the age of generative AI that we have not discussed but that you consider important? | Please feel free to elaborate on any aspect of your experience. |

**Appendix D**

**Participant Profiles**

| **Participant** | **Discipline** | **Academic Rank** | **Teaching Experience** | **Self-Reported AI Experience** | **Primary Uses of Generative AI** |
| --- | --- | --- | --- | --- | --- |
| P1 | Education | Assistant Professor | 11–15 years | Advanced | Assessment design, feedback |
| P2 | Medical & Health Sciences | Lecturer | 6–10 years | Intermediate | Clinical teaching, lesson preparation |
| P3 | Engineering & Technology | Associate Professor | More than 15 years | Advanced | Programming support, project supervision |
| P4 | Business & Economics | Assistant Professor | 6–10 years | Intermediate | Case-based learning, assessment |
| P5 | Humanities & Social Sciences | Professor | More than 15 years | Beginner | Lesson planning, instructional materials |
| P6 | Education | Associate Professor | 11–15 years | Advanced | Curriculum design, assessment |
| P7 | Medical & Health Sciences | Assistant Professor | 6–10 years | Intermediate | Clinical scenarios, student feedback |
| P8 | Engineering & Technology | Lecturer | 1–5 years | Intermediate | Project-based learning, coding assistance |
| P9 | Business & Economics | Professor | More than 15 years | Advanced | Assessment development, feedback |
| P10 | Humanities & Social Sciences | Assistant Professor | 11–15 years | Beginner | Course preparation, content generation |
| P11 | Education | Professor | More than 15 years | Advanced | Curriculum development, assessment |
| P12 | Medical & Health Sciences | Lecturer | 6–10 years | Intermediate | Learning support, feedback |
| P13 | Engineering & Technology | Assistant Professor | 11–15 years | Advanced | Design support, project supervision |
| P14 | Business & Economics | Associate Professor | 11–15 years | Intermediate | Teaching materials, case studies |
| P15 | Humanities & Social Sciences | Lecturer | 6–10 years | Beginner | Content preparation, brainstorming |
| P16 | Education | Assistant Professor | More than 15 years | Advanced | Teaching design, assessment |
| P17 | Medical & Health Sciences | Assistant Professor | 6–10 years | Intermediate | Clinical education, lesson planning |
| P18 | Engineering & Technology | Associate Professor | 11–15 years | Intermediate | Programming instruction, assessment |
| P19 | Business & Economics | Lecturer | 1–5 years | Beginner | Course preparation, instructional support |
| P20 | Medical & Health Sciences | Assistant Professor | 1–5 years | Intermediate | Writing support, discussion facilitation |
| P21 | Education | Assistant Professor | 6–10 years | Advanced | Reflective learning activities |
| P22 | Medical & Health Sciences | Assistant Professor | 1–5 years | Intermediate | Curriculum planning, clinical teaching |
| P23 | Medical & Health Sciences | Lecturer | 1–5 years | Beginner | Lesson preparation, student support |
